# 6-Benzyl 4-ethyl 2-chloro-5,6,7,8-tetra­hydro­pyrido[4,3-*d*]pyrimidine-4,6-di­carboxyl­ate

**DOI:** 10.1107/S1600536811034313

**Published:** 2011-08-27

**Authors:** Huan-Mei Guo

**Affiliations:** a Microscale Science Institute, Weifang University, Weifang 261061, People’s Republic of China

## Abstract

In the title compound, C_18_H_18_ClN_3_O_4_, the dihedral angle between the pyrimidine ring and the N-bonded ester grouping is 56.27 (7)° and the dihedral angle between the aromatic rings is 11.23 (7)°.

## Related literature

For background to the biological activities of pyrimidine compounds, see: Patil *et al.* (2003 ▶); Siddiqui *et al.* (2007 ▶).
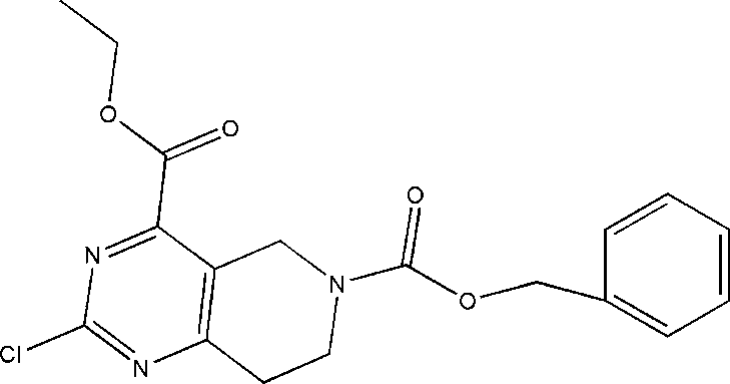


## Experimental

### 

#### Crystal data

C_18_H_18_ClN_3_O_4_
*M* *_r_* = 375.80
Monoclinic, 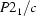

*a* = 11.530 (2) Å
*b* = 12.384 (2) Å
*c* = 14.010 (3) Å
β = 119.820 (4)°
*V* = 1735.6 (6) Å^3^
*Z* = 4
Mo *K*α radiation
μ = 0.25 mm^−1^
*T* = 173 K
0.23 × 0.20 × 0.16 mm

#### Data collection

MM007-HF CCD (Saturn 724+) diffractometer
Absorption correction: multi-scan (*CrystalClear*; Rigaku, 2007 ▶) *T*_min_ = 0.945, *T*_max_ = 0.961
8909 measured reflections
3925 independent reflections
3518 reflections with *I* > 2σ(*I*)
*R*_int_ = 0.038

#### Refinement

*R*[*F* ^2^ > 2σ(*F* ^2^)] = 0.058
*wR*(*F* ^2^) = 0.142
*S* = 1.09
3925 reflections
236 parameters
H-atom parameters constrained
Δρ_max_ = 0.65 e Å^−3^
Δρ_min_ = −0.70 e Å^−3^

### 

Data collection: *CrystalClear* (Rigaku, 2007 ▶); cell refinement: *CrystalClear*; data reduction: *CrystalClear*; program(s) used to solve structure: *SHELXS97* (Sheldrick, 2008 ▶); program(s) used to refine structure: *SHELXL97* (Sheldrick, 2008 ▶); molecular graphics: *SHELXTL* (Sheldrick, 2008 ▶); software used to prepare material for publication: *SHELXTL*.

## Supplementary Material

Crystal structure: contains datablock(s) global, I. DOI: 10.1107/S1600536811034313/hb6350sup1.cif

Structure factors: contains datablock(s) I. DOI: 10.1107/S1600536811034313/hb6350Isup2.hkl

Supplementary material file. DOI: 10.1107/S1600536811034313/hb6350Isup3.cml

Additional supplementary materials:  crystallographic information; 3D view; checkCIF report

## Figures and Tables

**Fig. 1.**
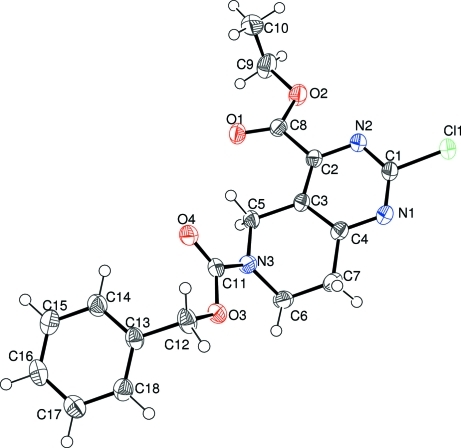
The molecular structure of (I) with displacement ellipsoids drawn at the 50% probability level.

## supplementary crystallographic information

### Comment

Pyrimidine is a widespread heterocyclic moiety present in numerous natural products. Pyrimidines are important not only because they are an integral part of genetic materials, but also they have important biodynamic properties and biological activities (e.g. Siddiqui *et al.* (2007); Patil *et al.* (2003).

In continuation of our interest in the synthesis of the biologically active heterocyclic compound, here we report the sigle crystal structure of the title compound, (I).  In the molecule (Fig. 1), atoms N1,C1,N2,C2,C3 and C4 lie in a plane (p1), with a maximum deviation of 0.0312 (13) Å, atoms N3,C11,O3,O4, and C12 lie in a plane (p2) too, the maximum deviation is 0.0647 (13) Å. The dihedral angle between p1 and p2 is 56.27 (7)°. The dihedral angles made by the phenyl ring with p1 and p2 are 11.23 (7)° and 60.25 (8)°, respectively.

### Experimental

5-*tert*-butyl 3-ethyl 1-isopropyl-6,7-dihydro-1*H*-pyrazolo[4,3-*c*] pyridine-3,5(4*H*)-dicarboxylate was synthesized with 6-benzyl 4-ethyl 2-hydroxy-7,8-dihydropyrido[4,3-*d*] pyrimidine-4,6(5*H*)- dicarboxylate (1 eq), andN,*N*-dimethylaniline (2 eq) in POCl3 (as solvent) in refluxing for 3 hrs. Colourless blocks of (I) were obtained by recrystallization from ethanol at room temperature.

### Refinement

All H atoms were fixed geometrically and allowed to ride on their attached atoms, with C—H distances in the range 0.95–0.98 Å, and with *U*_iso_(H) = 1.2*U*_eq_(C) or *U*_iso_(H) = 1.5*U*_eq_(C_methyl_).

### Crystal data

**Table:**

C_18_H_18_ClN_3_O_4_	*F*(000) = 784
*M**_r_* = 375.80	*D*_x_ = 1.438 Mg m^−^^3^
Monoclinic, *P*2_1_/*c*	Mo *K*α radiation, λ = 0.71073 Å
Hall symbol: -P 2ybc	Cell parameters from 6088 reflections
*a* = 11.530 (2) Å	θ = 1.6–27.5°
*b* = 12.384 (2) Å	µ = 0.25 mm^−^^1^
*c* = 14.010 (3) Å	*T* = 173 K
β = 119.820 (4)°	Block, colorless
*V* = 1735.6 (6) Å^3^	0.23 × 0.20 × 0.16 mm
*Z* = 4	

### Data collection

**Table:**

MM007-HF CCD (Saturn 724+) diffractometer	3925 independent reflections
Radiation source: rotating anode	3518 reflections with *I* > 2σ(*I*)
Confocal	*R*_int_ = 0.038
ω scans at fixed χ = 45°	θ_max_ = 27.5°, θ_min_ = 2.0°
Absorption correction: multi-scan (*CrystalClear*; Rigaku, 2007)	*h* = −14→12
*T*_min_ = 0.945, *T*_max_ = 0.961	*k* = −12→16
8909 measured reflections	*l* = −16→18

### Refinement

**Table:**

Refinement on *F*^2^	Primary atom site location: structure-invariant direct methods
Least-squares matrix: full	Secondary atom site location: difference Fourier map
*R*[*F*^2^ > 2σ(*F*^2^)] = 0.058	Hydrogen site location: inferred from neighbouring sites
*wR*(*F*^2^) = 0.142	H-atom parameters constrained
*S* = 1.09	*w* = 1/[σ^2^(*F*_o_^2^) + (0.0565*P*)^2^ + 1.1725*P*] where *P* = (*F*_o_^2^ + 2*F*_c_^2^)/3
3925 reflections	(Δ/σ)_max_ < 0.001
236 parameters	Δρ_max_ = 0.65 e Å^−^^3^
0 restraints	Δρ_min_ = −0.70 e Å^−^^3^

### Special details

**Table:**

Geometry. All e.s.d.'s (except the e.s.d. in the dihedral angle between two l.s. planes) are estimated using the full covariance matrix. The cell e.s.d.'s are taken into account individually in the estimation of e.s.d.'s in distances, angles and torsion angles; correlations between e.s.d.'s in cell parameters are only used when they are defined by crystal symmetry. An approximate (isotropic) treatment of cell e.s.d.'s is used for estimating e.s.d.'s involving l.s. planes.
Refinement. Refinement of *F*^2^ against ALL reflections. The weighted *R*-factor *wR* and goodness of fit *S* are based on *F*^2^, conventional *R*-factors *R* are based on *F*, with *F* set to zero for negative *F*^2^. The threshold expression of *F*^2^ > σ(*F*^2^) is used only for calculating *R*-factors(gt) *etc*. and is not relevant to the choice of reflections for refinement. *R*-factors based on *F*^2^ are statistically about twice as large as those based on *F*, and *R*- factors based on ALL data will be even larger.

### Fractional atomic coordinates and isotropic or equivalent isotropic displacement parameters (Å^2^)

**Table:**

	*x*	*y*	*z*	*U*_iso_*/*U*_eq_	
Cl1	0.70799 (5)	0.21924 (5)	0.19746 (6)	0.0470 (2)	
O1	0.11289 (14)	0.13056 (12)	0.00647 (13)	0.0367 (4)	
O2	0.29267 (15)	0.02745 (12)	0.10881 (13)	0.0405 (4)	
O3	0.04486 (14)	0.60281 (12)	−0.14053 (12)	0.0329 (3)	
O4	−0.02266 (14)	0.43250 (12)	−0.20318 (12)	0.0349 (4)	
N1	0.51941 (17)	0.36384 (15)	0.13641 (14)	0.0320 (4)	
N2	0.45847 (16)	0.17871 (14)	0.12649 (14)	0.0299 (4)	
N3	0.12864 (17)	0.46466 (14)	−0.02346 (14)	0.0316 (4)	
C1	0.5421 (2)	0.25868 (18)	0.14647 (17)	0.0315 (4)	
C2	0.33018 (19)	0.20875 (16)	0.08209 (15)	0.0256 (4)	
C3	0.28806 (18)	0.31618 (16)	0.05975 (14)	0.0246 (4)	
C4	0.3906 (2)	0.39277 (17)	0.09374 (16)	0.0280 (4)	
C5	0.14314 (19)	0.35043 (16)	0.00482 (16)	0.0282 (4)	
H5A	0.0904	0.3072	−0.0627	0.034*	
H5B	0.1076	0.3363	0.0550	0.034*	
C6	0.2223 (2)	0.53565 (17)	0.06382 (17)	0.0353 (5)	
H6A	0.2174	0.5234	0.1315	0.042*	
H6B	0.1992	0.6120	0.0415	0.042*	
C7	0.3628 (2)	0.51207 (17)	0.08584 (19)	0.0365 (5)	
H7A	0.3741	0.5431	0.0258	0.044*	
H7B	0.4284	0.5473	0.1555	0.044*	
C8	0.2320 (2)	0.11846 (16)	0.06036 (16)	0.0280 (4)	
C9	0.2049 (2)	−0.0631 (2)	0.0968 (2)	0.0466 (6)	
H9A	0.1219	−0.0353	0.0924	0.056*	
H9B	0.2500	−0.1104	0.1623	0.056*	
C10	0.1705 (3)	−0.1273 (2)	−0.0041 (2)	0.0520 (7)	
H10A	0.1185	−0.0824	−0.0694	0.078*	
H10B	0.1175	−0.1905	−0.0072	0.078*	
H10C	0.2528	−0.1511	−0.0019	0.078*	
C11	0.04534 (18)	0.49442 (17)	−0.12869 (16)	0.0279 (4)	
C12	−0.0290 (2)	0.6427 (2)	−0.25290 (18)	0.0364 (5)	
H12A	−0.0266	0.5874	−0.3030	0.044*	
H12B	0.0157	0.7083	−0.2592	0.044*	
C13	−0.1726 (2)	0.66913 (17)	−0.28908 (16)	0.0293 (4)	
C14	−0.2715 (2)	0.59019 (18)	−0.32842 (17)	0.0343 (5)	
H14	−0.2482	0.5171	−0.3310	0.041*	
C15	−0.4040 (2)	0.6170 (2)	−0.36400 (18)	0.0390 (5)	
H15	−0.4707	0.5624	−0.3894	0.047*	
C16	−0.4380 (2)	0.7237 (2)	−0.36211 (18)	0.0388 (5)	
H16	−0.5285	0.7426	−0.3874	0.047*	
C17	−0.3409 (2)	0.8033 (2)	−0.32367 (18)	0.0370 (5)	
H17	−0.3647	0.8766	−0.3228	0.044*	
C18	−0.2084 (2)	0.77566 (18)	−0.28631 (17)	0.0321 (4)	
H18	−0.1416	0.8302	−0.2586	0.039*	

### Atomic displacement parameters (Å^2^)

**Table:**

	*U*^11^	*U*^22^	*U*^33^	*U*^12^	*U*^13^	*U*^23^
Cl1	0.0225 (3)	0.0441 (3)	0.0666 (4)	−0.0014 (2)	0.0163 (3)	−0.0168 (3)
O1	0.0234 (7)	0.0309 (8)	0.0474 (9)	−0.0025 (6)	0.0112 (6)	0.0032 (7)
O2	0.0296 (8)	0.0273 (8)	0.0499 (9)	−0.0021 (6)	0.0085 (7)	0.0081 (7)
O3	0.0283 (7)	0.0276 (7)	0.0328 (7)	0.0048 (6)	0.0077 (6)	0.0067 (6)
O4	0.0286 (7)	0.0335 (8)	0.0307 (7)	0.0053 (6)	0.0057 (6)	−0.0014 (6)
N1	0.0244 (8)	0.0322 (9)	0.0354 (9)	−0.0054 (7)	0.0118 (7)	−0.0068 (7)
N2	0.0231 (8)	0.0311 (9)	0.0313 (8)	−0.0004 (7)	0.0103 (7)	−0.0049 (7)
N3	0.0283 (9)	0.0249 (9)	0.0290 (8)	0.0018 (7)	0.0047 (7)	0.0018 (7)
C1	0.0221 (9)	0.0352 (11)	0.0329 (10)	−0.0015 (8)	0.0105 (8)	−0.0072 (9)
C2	0.0228 (9)	0.0274 (10)	0.0226 (8)	−0.0002 (7)	0.0083 (7)	−0.0005 (7)
C3	0.0222 (9)	0.0273 (9)	0.0212 (8)	−0.0019 (7)	0.0083 (7)	−0.0002 (7)
C4	0.0266 (9)	0.0289 (10)	0.0249 (9)	−0.0033 (8)	0.0100 (8)	−0.0025 (8)
C5	0.0223 (9)	0.0274 (10)	0.0291 (10)	0.0014 (8)	0.0083 (8)	0.0033 (8)
C6	0.0347 (11)	0.0264 (10)	0.0312 (10)	0.0027 (8)	0.0061 (9)	−0.0025 (8)
C7	0.0314 (11)	0.0259 (10)	0.0397 (12)	−0.0054 (9)	0.0084 (9)	−0.0023 (9)
C8	0.0268 (10)	0.0259 (10)	0.0278 (9)	−0.0012 (8)	0.0110 (8)	0.0004 (7)
C9	0.0371 (12)	0.0343 (12)	0.0531 (14)	−0.0074 (10)	0.0108 (11)	0.0146 (11)
C10	0.0436 (14)	0.0340 (13)	0.0746 (18)	−0.0014 (11)	0.0264 (13)	−0.0056 (12)
C11	0.0208 (9)	0.0292 (10)	0.0312 (10)	0.0027 (8)	0.0110 (8)	0.0012 (8)
C12	0.0275 (11)	0.0420 (13)	0.0359 (11)	0.0062 (9)	0.0130 (9)	0.0148 (9)
C13	0.0252 (9)	0.0332 (11)	0.0262 (9)	0.0037 (8)	0.0103 (8)	0.0092 (8)
C14	0.0298 (10)	0.0340 (11)	0.0331 (10)	0.0035 (9)	0.0110 (9)	0.0066 (9)
C15	0.0290 (11)	0.0460 (13)	0.0351 (11)	−0.0053 (10)	0.0107 (9)	0.0017 (10)
C16	0.0256 (10)	0.0527 (14)	0.0339 (11)	0.0078 (10)	0.0116 (9)	0.0047 (10)
C17	0.0362 (11)	0.0393 (12)	0.0343 (11)	0.0076 (10)	0.0167 (9)	0.0036 (9)
C18	0.0292 (10)	0.0344 (11)	0.0294 (10)	−0.0010 (9)	0.0120 (8)	0.0053 (8)

### Geometric parameters (Å, °)

**Table:**

Cl1—C1	1.746 (2)	C6—H6B	0.9900
O1—C8	1.203 (2)	C7—H7A	0.9900
O2—C8	1.322 (2)	C7—H7B	0.9900
O2—C9	1.463 (3)	C9—C10	1.493 (4)
O3—C11	1.352 (2)	C9—H9A	0.9900
O3—C12	1.454 (2)	C9—H9B	0.9900
O4—C11	1.216 (2)	C10—H10A	0.9800
N1—C1	1.322 (3)	C10—H10B	0.9800
N1—C4	1.344 (3)	C10—H10C	0.9800
N2—C1	1.312 (3)	C12—C13	1.508 (3)
N2—C2	1.341 (2)	C12—H12A	0.9900
N3—C11	1.350 (2)	C12—H12B	0.9900
N3—C5	1.456 (3)	C13—C18	1.389 (3)
N3—C6	1.456 (3)	C13—C14	1.391 (3)
C2—C3	1.397 (3)	C14—C15	1.392 (3)
C2—C8	1.510 (3)	C14—H14	0.9500
C3—C4	1.402 (3)	C15—C16	1.383 (3)
C3—C5	1.512 (3)	C15—H15	0.9500
C4—C7	1.504 (3)	C16—C17	1.383 (3)
C5—H5A	0.9900	C16—H16	0.9500
C5—H5B	0.9900	C17—C18	1.389 (3)
C6—C7	1.519 (3)	C17—H17	0.9500
C6—H6A	0.9900	C18—H18	0.9500
			
C8—O2—C9	115.82 (17)	O2—C9—C10	111.1 (2)
C11—O3—C12	115.75 (17)	O2—C9—H9A	109.4
C1—N1—C4	115.24 (17)	C10—C9—H9A	109.4
C1—N2—C2	114.46 (18)	O2—C9—H9B	109.4
C11—N3—C5	119.03 (17)	C10—C9—H9B	109.4
C11—N3—C6	125.44 (18)	H9A—C9—H9B	108.0
C5—N3—C6	114.90 (16)	C9—C10—H10A	109.5
N2—C1—N1	129.44 (19)	C9—C10—H10B	109.5
N2—C1—Cl1	114.57 (16)	H10A—C10—H10B	109.5
N1—C1—Cl1	115.97 (16)	C9—C10—H10C	109.5
N2—C2—C3	123.25 (18)	H10A—C10—H10C	109.5
N2—C2—C8	115.55 (17)	H10B—C10—H10C	109.5
C3—C2—C8	121.17 (17)	O4—C11—N3	124.76 (19)
C2—C3—C4	115.31 (18)	O4—C11—O3	124.00 (18)
C2—C3—C5	123.65 (17)	N3—C11—O3	111.23 (17)
C4—C3—C5	121.03 (18)	O3—C12—C13	113.06 (17)
N1—C4—C3	121.96 (19)	O3—C12—H12A	109.0
N1—C4—C7	116.26 (18)	C13—C12—H12A	109.0
C3—C4—C7	121.77 (18)	O3—C12—H12B	109.0
N3—C5—C3	111.06 (16)	C13—C12—H12B	109.0
N3—C5—H5A	109.4	H12A—C12—H12B	107.8
C3—C5—H5A	109.4	C18—C13—C14	118.86 (19)
N3—C5—H5B	109.4	C18—C13—C12	119.29 (19)
C3—C5—H5B	109.4	C14—C13—C12	121.8 (2)
H5A—C5—H5B	108.0	C13—C14—C15	120.8 (2)
N3—C6—C7	108.99 (18)	C13—C14—H14	119.6
N3—C6—H6A	109.9	C15—C14—H14	119.6
C7—C6—H6A	109.9	C16—C15—C14	119.5 (2)
N3—C6—H6B	109.9	C16—C15—H15	120.3
C7—C6—H6B	109.9	C14—C15—H15	120.3
H6A—C6—H6B	108.3	C15—C16—C17	120.4 (2)
C4—C7—C6	111.75 (18)	C15—C16—H16	119.8
C4—C7—H7A	109.3	C17—C16—H16	119.8
C6—C7—H7A	109.3	C16—C17—C18	119.8 (2)
C4—C7—H7B	109.3	C16—C17—H17	120.1
C6—C7—H7B	109.3	C18—C17—H17	120.1
H7A—C7—H7B	107.9	C13—C18—C17	120.7 (2)
O1—C8—O2	125.15 (19)	C13—C18—H18	119.7
O1—C8—C2	122.78 (18)	C17—C18—H18	119.7
O2—C8—C2	112.07 (16)		
			
C2—N2—C1—N1	5.3 (3)	C9—O2—C8—O1	−1.8 (3)
C2—N2—C1—Cl1	−176.77 (14)	C9—O2—C8—C2	177.02 (18)
C4—N1—C1—N2	−4.1 (3)	N2—C2—C8—O1	−168.70 (19)
C4—N1—C1—Cl1	177.96 (14)	C3—C2—C8—O1	13.1 (3)
C1—N2—C2—C3	−0.7 (3)	N2—C2—C8—O2	12.5 (2)
C1—N2—C2—C8	−178.82 (17)	C3—C2—C8—O2	−165.70 (18)
N2—C2—C3—C4	−4.1 (3)	C8—O2—C9—C10	89.1 (3)
C8—C2—C3—C4	173.94 (17)	C5—N3—C11—O4	1.8 (3)
N2—C2—C3—C5	177.00 (18)	C6—N3—C11—O4	172.3 (2)
C8—C2—C3—C5	−5.0 (3)	C5—N3—C11—O3	−179.43 (17)
C1—N1—C4—C3	−1.7 (3)	C6—N3—C11—O3	−9.0 (3)
C1—N1—C4—C7	177.53 (19)	C12—O3—C11—O4	−8.4 (3)
C2—C3—C4—N1	5.3 (3)	C12—O3—C11—N3	172.86 (17)
C5—C3—C4—N1	−175.74 (18)	C11—O3—C12—C13	91.6 (2)
C2—C3—C4—C7	−173.87 (18)	O3—C12—C13—C18	98.8 (2)
C5—C3—C4—C7	5.1 (3)	O3—C12—C13—C14	−83.2 (3)
C11—N3—C5—C3	124.66 (19)	C18—C13—C14—C15	−0.3 (3)
C6—N3—C5—C3	−46.7 (2)	C12—C13—C14—C15	−178.33 (19)
C2—C3—C5—N3	−170.82 (17)	C13—C14—C15—C16	1.3 (3)
C4—C3—C5—N3	10.3 (3)	C14—C15—C16—C17	−1.0 (3)
C11—N3—C6—C7	−104.5 (2)	C15—C16—C17—C18	−0.3 (3)
C5—N3—C6—C7	66.2 (2)	C14—C13—C18—C17	−1.0 (3)
N1—C4—C7—C6	−165.93 (18)	C12—C13—C18—C17	177.13 (19)
C3—C4—C7—C6	13.3 (3)	C16—C17—C18—C13	1.2 (3)
N3—C6—C7—C4	−46.0 (2)		

## References

[bb1] Patil, L. R., Mandhare, P. N., Bondge, S. P., Munde, S. B. & Mane, R. (2003). *Indian J. Heterocycl. Chem.* **12**, 245–248.

[bb2] Rigaku (2007). *CrystalClear* Rigaku Corporation, Tokyo, Japan.

[bb3] Sheldrick, G. M. (2008). *Acta Cryst.* A**64**, 112–122. 10.1107/S0108767307043930 18156677

[bb4] Siddiqui, A. A., Rajesh, R. Mojahid-Ul-Islam, Alagarsamy, V., Meyyanathan, S. N., Kumar, B. P. & Suresh, B. (2007). *Acta Pol. Pharm.* **64**, 17–26. 17665846

